# Flexible Regulation of Positive and Negative Emotion Expression: Reexamining the Factor Structure of the Flexible Regulation of Emotional Expression Scale (FREE) Based on Emotion Valence

**DOI:** 10.3390/jintelligence12090085

**Published:** 2024-09-02

**Authors:** Yanhua Zhao, Ping Wang

**Affiliations:** 1 School of Psychology, Henan University, Jinming Campus, Kaifeng 475001, China; yz@vip.henu.edu.cn; 2 Center for Teacher Education, School of Education Sciences, Henan University, Jinming Campus, Kaifeng 475001, China

**Keywords:** expression regulation, flexibility, enhancement, suppression, positive and negative emotions, mental health, relationship stress

## Abstract

The Flexible Regulation of Emotional Expression (FREE) Scale assesses individuals’ ability to enhance and suppress their emotional expressions across different situations. This study investigates the optimal factor structure of the FREE and emphasizes the importance of distinguishing between the regulation abilities for positive and negative expressions. A sample of 607 undergraduates (Mage = 19.02, SD = 1.02, 72.2% female) from Mainland China completed the questionnaire survey. Confirmatory factor analyses tested eight competing models of the FREE structure. Results indicated that the second-order model, featuring two higher-order factors (expressive enhancement and suppression abilities), fit the data well. An alternative second-order model, with two different higher-order factors (positive and negative emotion expressive abilities) and the same four first-order factors, demonstrated an even better fit. Various types of expressive ability scores showed predictive validity regarding emotion regulation self-efficacy, mental health outcomes, and relationship stress. Regulation of emotional expression can be represented by either regulation type or emotion valence, with the latter providing more informative insights. Flexible regulation of positive and negative emotion expression predicted fewer symptoms of depression, anxiety, stress, and relationship stress beyond emotion regulation self-efficacy. These findings highlight the importance of considering emotional valence in understanding flexibility in expression regulation.

## 1. Introduction

Emotion regulation flexibility significantly influences psychological adjustment (Aldao and Nolen-Hoeksema 2012; Bonanno et al. 2004; Sheppes et al. 2014). The concept of regulatory flexibility in the field of emotional expression has enhanced our understanding of the expression regulation process. Expression regulation ability refers to individual differences in the ability to both enhance (up-regulate) and suppress (down-regulate) emotional expressions (Burton and Bonanno 2016). The ability to enhance (enhancement) and suppress (suppression) emotions, as well as the flexibility in using these abilities according to the context, can lead to different outcomes (Bonanno and Burton 2013). Previous research has demonstrated that individual differences in regulation abilities and flexibility in emotional expression have distinct impacts on mental health outcomes and social functioning (Chen et al. 2018; Gonzalez-Escamilla et al. 2022; Pollastri et al. 2018; Quattropani et al. 2022; Wang and Hawk 2019). This distinction highlights the importance of understanding how adaptive expression regulation strategies and their flexible application can contribute to psychological and social functioning.

The primary methods for studying expression regulation flexibility include laboratory experiments and questionnaire-based approaches. Early research predominantly used experimental methods to investigate the impact of expressive flexibility on specific tasks, providing insights into the causal relationship between emotion regulation flexibility and key outcomes (Bonanno et al. 2004; Westphal et al. 2010). However, due to the limitations of experimental methods in exploring the broader and long-term effects of expressive flexibility, Burton and Bonanno (2016) developed a reliable and concise questionnaire to measure this construct. The questionnaire, known as the Flexible Regulation of Emotional Expression (FREE), comprises 16 items and imposes a minimal response burden on participants, making it suitable for use in large and diverse samples as well as longitudinal research designs.

### 1.1. Factor Structure of FREE

The FREE was originally conceptualized as a multi-dimensional tool, with its structure defined by four dimensions derived from the up- and down-regulation of positive and negative emotions: enhancement of positive emotion, enhancement of negative emotion, suppression of positive emotion, and suppression of negative emotion. Burton and Bonanno (2016) identified a second-order factor model with enhancement and suppression abilities as hierarchical factors as the best representation of the FREE scale’s structure. This model suggested that enhancement and suppression abilities, along with their flexibility, warrant further investigation. Subsequent studies have supported this second-order factor structure in both adult samples (Chen et al. 2018; Gonzalez-Escamilla et al. 2022; Quattropani et al. 2022) and younger populations from various cultures (Haag et al. 2022; Wang and Hawk 2020).

However, previous research underscores the importance of distinguishing between the regulation of positive and negative emotions (Preece et al. 2018; Tsujimoto et al. 2024). Identifying only the regulation type (enhancement or suppression) without differentiating between positive and negative emotion expression can obscure their distinct effects. Therefore, it is crucial to separately address positive and negative expression regulation abilities and their respective flexibilities. Positive emotion expression regulation involves managing positive expressions through enhancement and suppression strategies, while negative emotion expression regulation pertains to managing negative emotions with similar strategies. Burton and Bonanno (2016) explored positive and negative valence dimensions using correlated two-factor models but did not use a hierarchical structure to differentiate between positive and negative expression abilities. Similarly, studies by Chen et al. (2018), Gonzalez-Escamilla et al. (2022), and Quattropani et al. (2022) followed this approach without employing a hierarchical framework for conceptualizing positive and negative expression regulation abilities. In contrast, our study estimates a second-order factor model that includes positive and negative expression regulation abilities as higher-order factors alongside other competing models. Additionally, following the methodology suggested by Burton and Bonanno (2016), we separately calculated the flexibility of positive and negative emotion expression.

### 1.2. Expression Regulation Abilities for Mental Health and Social Functioning

The role of expressive flexibility in mental health outcomes and social functioning has garnered significant attention in recent years. As a key component of emotional regulation, expressive flexibility is closely linked to better mental health outcomes, including but not limited to reductions in depression, anxiety, and stress symptoms (Chen et al. 2018; Gonzalez-Escamilla et al. 2022; Haag et al. 2022; Lenzo et al. 2021; Shangguan et al. 2022), and greater life satisfaction (Ang and Tsai 2023; Chen et al. 2018). The association between expressive regulation flexibility and mental health outcomes can be observed both momentarily (Chen et al. 2024) and longitudinally (Ang and Tsai 2023), and it spans across adult, child, and adolescent groups (Wang and Hawk 2020; Zhang et al. 2023). Furthermore, previous studies highlight the interaction between an individual’s expression regulation repertoire and contextual factors in explaining depression and anxiety (Chen and Bonanno 2021). In the social domain, expressive regulation flexibility significantly impacts social functioning. Higher expressive flexibility is associated with better social adaptation (Pollastri et al. 2018), peer acceptance (Wang and Hawk 2019), perceived social support (Shangguan et al. 2022), and peer relationships (Wang and Hawk 2019), as well as reduced social anxiety (Westphal et al. 2010).

Regarding the specific contribution of expression regulation ability, previous research has shown inconsistent findings in predicting individual life outcomes. Some studies have found that enhancement is more important in predicting certain aspects of life outcomes (Maccallum et al. 2021; Rodin et al. 2017), while others emphasize the greater importance of suppression (Chen et al. 2018; Westphal et al. 2010). Additionally, some studies have found that both abilities are important in predicting individuals’ mental health outcomes (Gonzalez-Escamilla et al. 2022; Gupta and Bonanno 2011; Wang and Hawk 2019). Using an adult sample from Italy, Quattropani et al. (2022) found that enhancement ability was positively associated with perceived mental health. However, regression analyses that controlled for age, gender, and emotional intelligence indicated that higher suppression ability and lower enhancement ability predicted better mental health. These inconsistencies may be closely related to the cultural environment in which individuals reside, as expressive flexibility may impact psychological outcomes differently across cultures (Ang and Tsai 2023; Strickland and Skolnick 2020). Another possibility for these inconsistencies might be that the influence of expression regulation abilities on mental health outcomes varies depending on whether positive or negative emotion expressions are being regulated.

### 1.3. Need to Differentiate between Positive and Negative Emotion Expression Abilities

Positive and negative emotion regulation are crucial predictors of mental health (Preece et al. 2018). Previous research has highlighted the differences between positive and negative emotion regulation abilities in predicting individuals’ mental health and life satisfaction (Kearns and Creaven 2017; Tsujimoto et al. 2024; Young et al. 2019). Specifically, positive emotion regulation ability, the capacity to manage positive affect, has been linked to higher life satisfaction and social support (Doorley and Kashdan 2021; Livingstone and Srivastava 2012), as well as lower loneliness and depression (Kearns and Creaven 2017; van Kleef et al. 2022). Conversely, negative emotion regulation ability—the capacity to effectively manage negative emotions—has been associated with better mental and social functioning (Aldao et al. 2010; Kearns and Creaven 2017; Saxena et al. 2011). Therefore, combining the enhancement or suppression abilities of positive and negative emotion expressions into a composite score may obscure their distinct effects, potentially leading to inconsistent results in predicting individual outcomes. To support the predictive validity of the FREE scale and emphasize the importance of differentiating the regulation of positive and negative emotion expressions, this study examined the associations between four expressive abilities (enhancement and suppression abilities, positive and negative abilities) and mental health outcomes (symptoms of depression, anxiety, and stress) and perceived relationship stress.

### 1.4. The Present Study

This study aimed to investigate the optimal factor structure of the FREE scale and highlight the importance of distinguishing between the regulation of positive and negative emotion expressions. Confirmatory factor analyses were conducted to estimate and compare several competing models of the FREE scale, and the reliability of different ability constructs was assessed. Additionally, the predictive validity of the FREE scale was evaluated by examining the associations between various types of expressive regulation abilities and flexibility with emotion regulation self-efficacy, mental health outcomes, and relationship stress.

## 2. Materials and Methods

### 2.1. Participants and Procedure

A total of 607 undergraduate students from two comprehensive universities in Mainland China participated in this study. Participants ranged in age from 16 to 23 years (M = 19.02, SD = 1.02), with the majority being female (72.2%, *n* = 425). In terms of socio-economic status (scaled from 1 for lowest to 10 for highest), 67.6% of participants reported their family background as middle level or below (M = 4.89, SD = 1.42). Demographically, 94.3% of participants were Han Chinese, while 5.7% belonged to various ethnic minorities. Geographically, 2.8% of participants were from the local city, 22.3% from other cities within the same province, and 74.9% from cities outside the province, with 6.1% not reporting their location. The study was conducted in a single session during an Introduction to Psychology class. All participants were fully informed about the study’s purpose and provided written consent before participating. They completed a questionnaire package that included all the measures detailed below. Upon submission of their questionnaires, participants received stationery gifts as incentives for their participation.

### 2.2. Measures

#### 2.2.1. The Flexible Regulation of Emotional Expression (FREE) Scale

The ability to modulate emotional expression was assessed using the Flexible Regulation of Emotional Expression (FREE) scale, developed by Burton and Bonanno (2016). This 16-item self-report measure evaluates four dimensions of emotion regulation: enhancing positive emotion (e.g., “A friend wins an award for a sport that doesn’t interest you”; 4 items), enhancing negative emotion (e.g., “Your friend is telling you about what a terrible day they had”; 4 items), suppressing positive emotion (e.g., “While having dinner with a friend who has just recently lost their job, you receive a phone call from your boss stating you will get a raise”; 4 items), and suppressing negative emotion (e.g., “You are at a social event and the person you’re talking to frequently spits while they speak”; 4 items). Each item is rated on a 6-point scale indicating how often participants believe each item pertains to them (1 = unable to 6 = very able). Certain FREE items are reverse-scored so that higher scores consistently indicate greater flexibility in emotion regulation. The FREE scale has demonstrated good validity and reliability within Chinese samples. The original FREE scale provides three scores: enhancement ability, suppression ability, and expressive flexibility (EF). In accordance with the guidelines provided by Burton and Bonanno (2016), we calculated the scores as follows: (1) a sum score was obtained by adding the enhancement and suppression scores; (2) a polarity score was determined by calculating the absolute value of the difference between the enhancement and suppression scores; (3) EF was computed by subtracting the polarity score from the sum score. Thus, the FREE scale yields three scores: enhancement ability, suppression ability, and expressive flexibility, with higher scores indicating greater flexibility in regulating emotional expressions. The internal consistency of the scale was satisfactory, with Cronbach’s alpha coefficients of 0.81 for enhancement ability and 0.70 for suppression ability.

#### 2.2.2. Emotion Regulation Self-Efficacy

Regulatory emotion self-efficacy was assessed using the Chinese version of the Regulatory Emotion Self-Efficacy Scale (CRESE), developed by Wang et al. (2013), based on the framework established by Caprara et al. (2008). The CRESE evaluates an individual’s confidence in both expressing positive emotions and managing negative emotions. For this study, we focused on the items related to self-efficacy in managing negative emotions. Each item was rated on a 5-point Likert scale, with responses ranging from 1 (not at all true of me) to 5 (completely true of me). Higher scores reflect greater self-efficacy in regulating negative emotions. The scale demonstrated good reliability, with a Cronbach’s alpha coefficient of 0.86.

#### 2.2.3. Symptoms of Depressive, Anxiety, and Stress

The Chinese Version of the Depression, Anxiety, and Stress Scale (DASS-21) was used to assess negative emotional experiences across three domains: depression, anxiety, and stress (Gong et al. 2010). The DASS-21 comprises 21 items, with each subscale containing 7 items. Responses are rated on a 4-point Likert scale: 0 (did not apply to me at all) to 3 (applied to me very much or most of the time). In this study, the Cronbach’s alpha coefficients for the depression, anxiety, and stress subscales were 0.77, 0.79, and 0.76, respectively. The Cronbach’s alpha for the total scale was 0.89.

#### 2.2.4. College Students Stress

The College Stress Scale (CSSS), developed by Li and Mei (2002), measures the types and levels of stress experienced by college students in recent months. The CSSS assesses stress across three dimensions: personal stress, academic stress, and negative life events. For this study, we utilized the 16-item personal relationship stress subscale to specifically evaluate students’ stress related to their interpersonal relationships. The scale uses a 4-point Likert scale, where responses range from 0 (no stress) to 3 (extremely stressful). Higher scores indicate greater levels of stress. In this study, the Cronbach’s α for the relationship stress subscale was 0.84.

### 2.3. Data Analyses

First, we conducted a Confirmatory Factor Analysis (CFA) to determine the optimal model fit. Using Mplus 7.4, we assessed the original second-order factor model (where enhancement and suppression abilities serve as two higher-order factors) alongside several competing models. Model fit was evaluated using several widely accepted indices: the Comparative Fit Index (CFI), the Tucker–Lewis Index (TLI), the Root Mean Square Error of Approximation (RMSEA), the Standardized Root Mean Square Residual (SRMR), the Akaike Information Criterion (AIC), and the Bayesian Information Criterion (BIC). Following Hu and Bentler’s (1999) criteria, a CFI and TLI value of 0.95 or higher indicates a good fit, while an RMSEA and SRMR value of 0.06 or lower reflects a good fit. The AIC and BIC are employed to compare two or more non-nested models, with the lowest value indicating the best fit for the hypothesized model. Based on Cheung and Rensvold’s (2002) guidelines, a meaningful decrement in model fit was indicated by a CFI decrease greater than 0.01 and an RMSEA increase greater than 0.015. Second, we performed correlation and hierarchical multiple regression analyses to explore how enhancement and suppression abilities, as well as their flexibility, relate to mental health outcomes and relationship stress. Third, we conducted additional correlation and regression analyses to examine the associations between positive and negative emotion expression regulation abilities and their flexibility with mental health outcomes and relationship stress. Gender, age, and emotion regulation self-efficacy were included as covariates in all regression models. Finally, we compared the enhancement and suppression approaches with the positive and negative expression regulation approaches from both ability and flexibility perspectives.

## 3. Results

### 3.1. Descriptive Statistics

During data collection, 0.216% of the data (21 values) were missing. We began by performing a missing data analysis, with Little’s MCAR test revealing that the data were not missing completely at random (MCAR). However, given the test’s sensitivity to data distribution and sample size (Enders 2010), we carefully inspected the missing data patterns and found no significant trends. Consequently, we employed Expectation Maximization (EM) estimation for imputation and compared the item means before and after imputation. The difference in means was minimal, with changes less than 0.01. Table 1 displays the means, standard deviations, and Cronbach’s alpha coefficients for the various dimensions of emotion expression regulation abilities.

### 3.2. Factor Analysis

**Figure 1:**
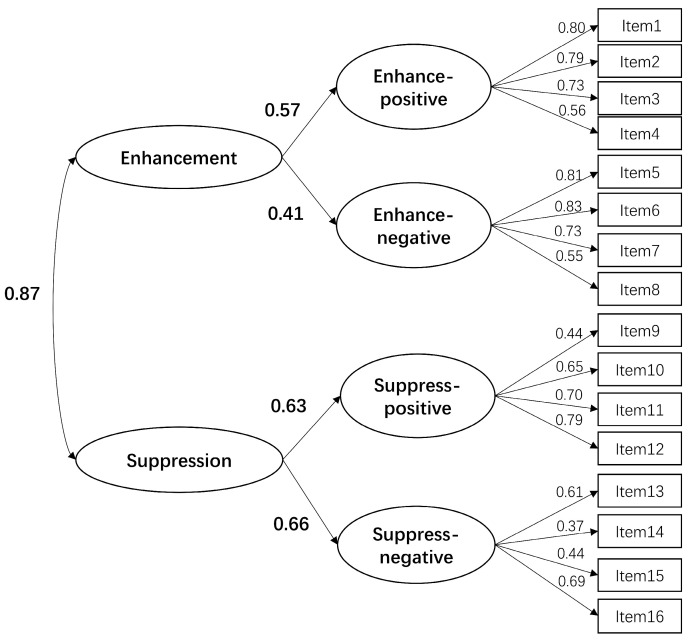
Standardized factor loadings for the second-order factor model with enhancement and suppression.

**Figure 2:**
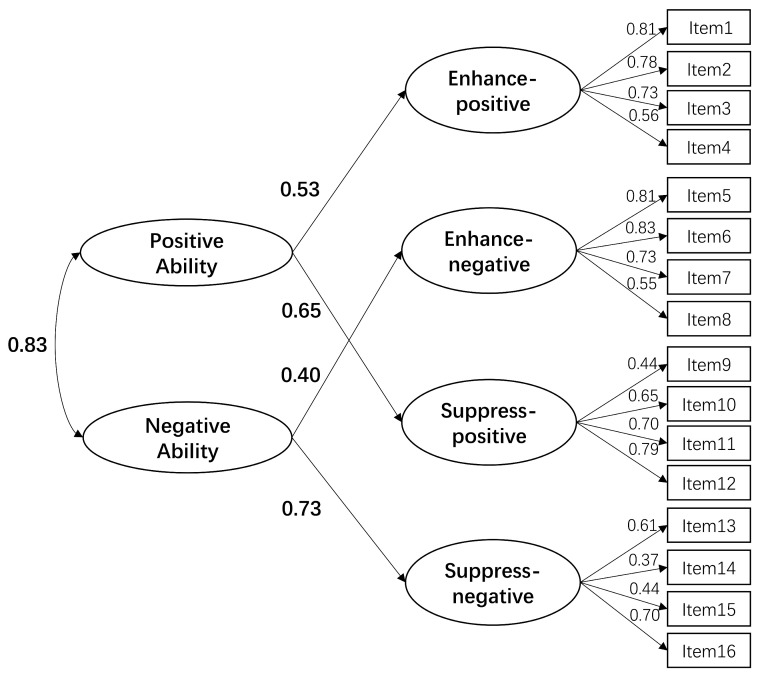
Standardized factor loadings for the second-order factor model with positive expressive ability and negative expressive ability.

Confirmatory factor analyses (CFAs) were performed to assess the factorial structure of the emotion regulation abilities as represented by the FREE scale (see Table 2). Several models were evaluated and compared using fit indices, including a one-factor model (M1) and two two-factor models: M2 (representing enhancement and suppression abilities) and M3 (representing positive and negative expression regulation abilities). None of these models exhibited adequate fit. The originally designed four-factor structure model (M4) demonstrated a good fit to the data (SBχ^2^(98) = 189.54, CFI = 0.958, RMSEA = 0.039 [90% CI: 0.031–0.048], SRMR = 0.050). Additionally, a second-order model (M5, see Figure 1), with two higher-order factors (enhancement and suppression abilities), also fit the data well (SBχ^2^(99) = 193.63, CFI = 0.956, RMSEA = 0.040 [90% CI: 0.031–0.048], SRMR = 0.051). Another proposed second-order model (M6, see Figure 2) with two different higher-order factors (positive and negative expression abilities) showed an excellent fit (SBχ^2^(99) = 193.07, CFI = 0.956, RMSEA = 0.040 [90% CI: 0.031–0.048], SRMR = 0.051, TLI = 0.947). According to the model change criteria suggested by Cheung and Rensvold (2002), M6 was not significantly different from M4 or M5, with changes in CFI and RMSEA smaller than the recommended thresholds.

Additionally, a hierarchical second-order model (M7) and a bifactor model (M8) were examined to explore the presence of a common factor underlying the four first-order factors. Model M7 presented a comparable fit (SBχ^2^(100) = 234.71, RMSEA = 0.047 [90% CI: 0.039–0.055], SRMR = 0.051, CFI = 0.951, TLI = 0.941). However, the enhancement of positive emotions did not load adequately on the hierarchical factor of expressive ability (λ = 0.019), indicating that treating expressive ability as a unidimensional construct may be inappropriate. The bifactor model (M8), with one general factor and four specific emotion regulation abilities, provided a significantly better fit (SBχ^2^(88) = 112.52, CFI = 0.989, TLI = 0.985, RMSEA = 0.021 [90% CI: 0.005–0.032], SRMR = 0.030). Despite this, the Explained Common Variance (ECV) for the general factor of expressive ability was 0.30, below the acceptable threshold of 0.50, and the composite reliability (McDonald’s Omega hierarchical) was 0.54, below the recommended cutoff of 0.70 (Rodriguez et al. 2016). Consequently, the four-factor model (M4) and the two second-order factor models (M5 and M6) were retained for further analysis.

### 3.3. Internal Consistency and Test–Rest Reliability

The reliability analyses (see Table 1) showed that the internal consistencies (Cronbach’s α) of the four subscales were good except suppress negative emotion: namely, enhance positive emotion (α = 0.81), enhance negative emotion (α = 0.82), suppress positive emotion (α = 0.73), and suppress negative emotion (α = 0.60). Whereas for the second-order factors (eight-item composites), namely enhancement ability (α = 0.77), suppression ability (α = 0.71), positive emotion ability (α = 0.78), and negative emotion ability (α = 0.71), the reliability estimates were well above 0.70. Overall, when considering all of the individual items, Cronbach’s α indicated good reliability of the FREE scale for emotional expression flexibility (α = 0.78; Table 1). According to the results of the internal consistencies (Cronbach’s α), the two higher-order factor models (M5 and M6) were retained for further analyses. The factor scores of these two models were saved and used for further regression analyses.

### 3.4. Predictive Validity

Table 1 presents the bivariate correlations between various dimensions of expression regulation abilities, flexibility, and other relevant variables. Emotion regulation self-efficacy showed significant positive correlations with all variables except for the enhancement ability of negative emotion. It was used as a control variable to examine the contributions of different approaches to structuring expressive ability and flexibility.

From an ability perspective, overall enhancement ability was negatively correlated with relationship stress (*p* < .01), while overall suppression ability was negatively correlated with anxiety, stress, and relationship stress (*p* < .05). To assess the predictive validity of these strategies, regression analyses were performed with the following model steps: gender and age as covariates (step 1), regulatory self-efficacy (step 2), enhancement and suppression abilities (step 3), and their interaction (step 4). As detailed in Table 3, the models accounted for 13% to 14% of the variance in mental health outcomes and 15% of the variance in relationship stress. Both enhancement and suppression strategies did not significantly predict mental health outcomes or relationship stress, contributing negligibly to the variance in health outcomes and relationship stress.

In contrast, positive expression regulation ability was negatively correlated with depression, anxiety, stress, and relationship stress (*p* < .01), while negative expression regulation ability showed no significant correlation with mental health outcomes or relationship stress. To evaluate the predictive validity of expressive abilities in relation to these two emotion dimensions, regression analyses were conducted, controlling for age, gender, and emotion regulation self-efficacy. Positive and negative expression regulation abilities and their interaction were entered sequentially. As shown in Table 4, the models explained 14% to 15% of the variance in mental health outcomes and 18% of the variance in relationship stress. Positive and negative expression regulation abilities significantly predicted both mental health outcomes and relationship stress, contributing an additional 2% to 3% of the variance in mental health outcomes and 3% of the variance in relationship stress. The emotion valence approach proved more effective in explaining the variance in mental health outcomes and relationship stress compared to the regulation-type approach.

From a flexibility perspective, flexibility in enhancing and suppressing emotions was negatively correlated with anxiety (*p* < .05), stress, and relationship stress (*p* < .01) and had a marginally negative correlation with depression (*p* < .10). To test the predictive validity of expressive flexibility (calculated based on the regulation type), regression analyses were conducted, controlling for age, gender, and regulatory self-efficacy. As shown in Table 5, the models explained 12% to 13% of the variance in mental health outcomes and 15% of the variance in relationship stress. Expressive flexibility did not significantly predict mental health outcomes or relationship stress, and no additional variance was explained by adding flexibility to the models.

However, when expressive flexibility was calculated separately for positive and negative emotions, the explained variance in mental health outcomes and relationship stress increased. Flexibility for positive expression regulation was negatively correlated with depression, anxiety, stress, and relationship stress (*p* < .01). Flexibility for negative expression regulation did not significantly correlate with mental health outcomes or relationship stress. To further test the predictive validity of expressive flexibility for positive and negative expressions, regression analyses were conducted, again controlling for age, gender, and regulatory self-efficacy. As shown in Table 6, the models explained 13% to 14% of the variance in mental health outcomes and 16% of the variance in relationship stress. Expressive flexibility for positive emotions significantly predicted relationship stress and marginally predicted mental health outcomes. Expressive flexibility for negative emotions significantly predicted symptoms of depression and marginally predicted symptoms of anxiety. Adding expressive flexibility for positive and negative emotions into the models explained approximately 1% additional variance. Overall, these findings indicate that higher ability and flexibility in expression regulation of positive emotions are generally associated with better mental health outcomes and lower relationship stress. Structuring the four expressive abilities using an emotion valence approach can explain more variance in mental health outcomes and relationship stress from both ability and flexibility perspectives.

## 4. Discussion

The present study examined the factor structure of the FREE scale, highlighting the importance of distinguishing between positive and negative expression regulation abilities. The findings support both the regulation-based higher-order factor model, which includes enhancement and suppression as composite abilities, and the emotion valence-based higher-order factor model, which includes positive and negative expression regulation as composite abilities. These results suggest that the ability to regulate emotion expression, as measured by the FREE scale, can be conceptualized either by regulation type (enhancement and suppression) or by emotional valence dimension (positive and negative emotions). Incorporating the emotional valence dimension provides a more informative interpretation of the ability to regulate emotional expressions.

Through CFA, this study examined and compared eight conceptual models. Consistent with previous research, the second-order factor model, which includes enhancement and suppression as higher-order abilities, demonstrated good fit indices and satisfactory reliability for both enhancement (α = 0.77) and suppression (α = 0.71). The four-factor model also showed good fit indices; however, the slightly lower reliability of the negative emotion suppression subscale (α = 0.60) raises questions about the separate use of the subscales. The other second-order factor model proposed in this study, which includes positive emotion expression regulation ability and negative emotion expression regulation ability as higher-order factors, also fits the data well. Although this model did not show significant differences in fit compared to the previous second-order model, the AIC and BIC indices indicated a slight advantage over the second-order model with enhancement and suppression abilities. Both positive emotion expression regulation ability (α = 0.78) and negative emotion expression regulation ability (α = 0.71) demonstrated satisfactory reliability. The one-factor models, two two-factor models, the second-order model (with overall emotion expression regulation ability as one higher-order factor), and the bifactor model (one global expressive ability factor and four specific ability factors) were not retained due to inadequate fit indices. These findings empirically support the necessity of differentiating between emotion dimensions and regulation types in studying expression regulation abilities.

Based on the correlation analysis, enhancement ability significantly predicted relationship stress but did not significantly correlate with mental health outcomes, consistent with previous research (Burton and Bonanno 2016; Chen et al. 2018). Suppression ability, however, was significantly correlated with mental health outcomes (with a marginally significant prediction for depression) and relationship stress, aligning with earlier studies (Burton and Bonanno 2016; Chen et al. 2018). Regarding emotion valences, positive emotion expression ability was significantly correlated with both mental health outcomes and relationship stress. These results are consistent with previous studies emphasizing the importance of positive emotion regulation for mental health and social functioning (Doorley and Kashdan 2021; Kearns and Creaven 2017; Park and Naragon-Gainey 2024; Preece et al. 2018). Unexpectedly, negative emotion expression regulation was not significantly correlated with mental health outcomes or relationship stress. One potential reason for this finding is that both enhancement and suppression of positive emotion expression were negatively correlated with mental health outcomes and relationship stress. In contrast, suppression of negative emotion expression was not significantly correlated with these outcomes, and enhancement of negative emotions was positively correlated with them. Thus, regardless of whether enhancement or suppression is used, the overall regulation of negative emotion expression does not significantly predict mental health outcomes or relationship stress. This finding contradicts earlier studies that emphasized the importance of negative emotion regulation (Aldao et al. 2010; Preece et al. 2018; Tsujimoto et al. 2024) but aligns with some results from Burton and Bonanno (2016). In Burton and Bonanno’s (2016) study, neither enhancement nor suppression of negative emotion expression was significantly correlated with social functioning, and enhancement of negative emotion expression was not significantly correlated with depression, with only suppression of negative emotion expression showing a significant correlation with depression. Therefore, when they combined the enhancement of positive emotion expression (a more influential factor) with the enhancement of negative emotion expression (a less influential factor) into an overall enhancement score, the significance of enhancement for mental health outcomes was no longer observed.

From a flexibility perspective, the overall flexibility of enhancement and suppression was less correlated with mental health outcomes and relationship stress compared to the flexibility of positive expression regulation. This discrepancy may be due to the lack of a significant correlation between the flexibility of negative emotion expression and these outcomes. When combining the abilities of enhancement and suppression for both positive and negative emotions, the overall correlation between emotion expression regulation flexibility and mental health outcomes and relationship stress decreased. These results highlight the need to separately examine the abilities and flexibility of positive and negative emotion expression regulation.

The regression analysis further underscores the necessity of distinguishing between positive and negative emotion expression abilities and their flexibility. After accounting for age, gender, and emotion regulation self-efficacy, neither enhancement nor suppression nor their interactions significantly predicted mental health outcomes or relationship stress. Adding regulation-based expressive abilities and their interactions did not significantly increase the explained variance in these outcomes. In contrast, incorporating positive and negative expression abilities based on emotion valences significantly enhanced the models’ ability to explain variance in mental health outcomes and relationship stress. Positive emotion expression ability significantly negatively predicted depression, anxiety (marginally significant), stress, and relationship stress, whereas negative emotion expression ability positively predicted these outcomes. Analysis from the perspective of flexibility revealed similar trends. Flexibility based on enhancement and suppression abilities did not significantly predict mental health outcomes or relationship stress and did not improve the models’ explanatory ability. However, distinguishing between positive and negative emotion expression flexibility showed that positive emotion expression flexibility significantly negatively predicted depression and stress (marginally significant) and reduced relationship stress. Conversely, negative emotion expression flexibility positively predicted increases in depression and anxiety, with the model explaining an additional 1% of the variance in mental health outcomes and relationship stress. This partially supports previous findings by Quattropani et al. (2022), which demonstrated a positive relationship between suppression ability and mental health outcomes. The adverse impact of regulating negative expressions on mental health may stem from the fact that both enhancement and suppression strategies may cause individuals to dwell on their negative emotions (Hamilton et al. 2011; Nolen-Hoeksema and Morrow 1993), potentially prolonging their adverse effects.

By differentiating between positive and negative emotion expression abilities and their flexibility, this study provides a more nuanced understanding of how these abilities influence mental health outcomes and social functioning. However, several limitations should be considered when interpreting the findings. First, the participants were predominantly college students with similar academic abilities and health statuses, which may not reflect the broader population from diverse backgrounds. Second, the sample was skewed toward females, potentially influencing the results, as gender can affect emotional regulation strategies and outcomes (Aldao et al. 2010). Future research should aim for a more balanced and diverse sample to enhance the generalizability of the results. Third, missing data imputation was performed in the current study, although the missing data were not missing completely at random (MCAR), which may have introduced some bias into the data analysis. Fourth, this study did not include items measuring regulatory efficacy for positive events. The lack of control for regulatory efficacy for positive emotions may limit the interpretation of the current results. However, even when regulatory self-efficacy for negative emotions was controlled for, the prediction of negative expressive ability for mental health outcomes and relationship stress remained significant. Furthermore, the regression analyses showed similar trends regardless of whether regulatory self-efficacy was controlled. Finally, compared to tools that assess emotion regulation abilities in predicting mental health outcomes (e.g., Difficulties in Emotion Regulation Scale; Zhao et al. 2022), FREE demonstrates relatively lower explanatory power for an individual’s mental health outcomes. This may be attributable to the limitations of the scenarios presented in the questionnaire (Burton and Bonanno 2016), which may not fully capture an individual’s capacity to flexibly regulate emotional expression across diverse situations. Additionally, from the perspective of the person–situation interaction model, the interpersonal and intrapersonal functions of expressive flexibility can vary depending on situational factors such as stress levels, relationships with others, or regulatory goals (Bonanno and Burton 2013). Given the importance of research on emotion regulation flexibility (Aldao and Nolen-Hoeksema 2012; Aldao et al. 2015; Bonanno and Burton 2013; Cheng et al. 2014), future studies should further investigate the roles of emotion expression flexibility in broader intrapersonal and interpersonal functioning.

## 5. Conclusions

This study supports the factor structure of the Flexible Regulation of Emotional Expression (FREE) scale and underscores the importance of considering both regulation-based and emotion type-based approaches to expression regulation ability and flexibility. The subscales for positive emotion expressive ability and negative emotion expressive ability demonstrated good construct validity, internal consistency, and predictive validity. Distinguishing between expressive abilities for positive and negative emotions provides better predictions for mental health outcomes and relationship stress compared to general enhancement and suppression abilities. Additionally, differentiating expressive flexibility in terms of positive and negative emotions offers greater explanatory power over general expressive flexibility. These findings underscore the importance of refining our understanding and assessment of expressive ability and flexibility to improve their application in mental health and social functioning contexts.

## Figures and Tables

**Table 1:** Means and correlations.

Variables	Mean	SD	α	Gender	Age	Regulatory Self-Efficacy	Depression	Anxiety	Stress	Relationship Stress
Enhance-positive	4.57	1.00	0.81	0.09 *	−0.08	0.27 **	−0.18 **	−0.16 **	−0.17 **	−0.25 **
Enhance-negative	3.35	1.08	0.82	−0.02	0.008	−0.03	0.09	0.09 *	0.09 *	0.06
Suppress-positive	4.18	1.03	0.73	0.01	−0.06	0.27 **	−0.14 **	−0.14 **	−0.16 **	−0.20 **
Suppress-negative	3.30	0.91	0.60	−0.11 *	0.03	0.22 **	0.02	−0.01	−0.04	−0.03
Enhancement	3.96	0.80	0.77	0.05	0.01	0.15 **	−0.05	−0.04	−0.04	−0.12 **
Suppression	3.74	0.77	0.71	−0.06	−0.02	0.31 **	−0.08 †	−0.09 *	−0.13 **	−0.15 **
Positive emotion expressive ability	4.37	0.82	0.78	0.06	−0.09	0.33 **	−0.19 **	−0.18 **	−0.20 **	−0.28 **
Negative emotion expressive ability	3.32	0.77	0.71	−0.07	0.08	0.11 **	0.07	0.06	0.04	0.02
Flexibility of enhancement and suppression	7.50	1.54	NA	−0.06	−0.02	0.31 **	−0.08 †	−0.09 *	−0.13 **	−0.15 **
Flexibility of positive emotion expression	8.39	2.06	NA	0.01	−0.06	0.27 **	−0.14 **	−0.14 **	−0.16 **	−0.21 **
Flexibility of negative emotion expression	6.61	1.82	NA	−0.11 *	0.03	0.22 **	0.02	−0.01	−0.04	−0.03
Total FREE score	3.85	0.64	0.78	−0.01	−0.01	0.28 **	−0.08 †	−0.08 †	−0.11 *	−0.16 **

Note. † *p* < .10, * *p* < .05, ** *p* < .01.

**Table 2:** Model fit.

	SBχ^2^	*df*	RMSEA (90% CI)	CFI	TLI	SRMR	AIC	BIC	Description
M1	2279.36	120	0.143 (0.136–0.150)	0.401	0.309	0.123	32,043.493	32,255.103	One-factor CFA
M2	1008.72	103	0.120 (0.114–0.127)	0.581	0.511	0.115	31,632.215	31,848.233	Two-factor CFA for enhance and suppression strategy ability
M3	760.61	103	0.103 (0.096–0.109)	0.695	0.645	0.107	31,338.621	31,399.076	Two-factor CFA for positive and negative ER ability
M4	189.54	98	0.039 (0.031–0.048)	0.958	0.948	0.050	30,658.216	30,896.276	Four-factor CFA for four subscales
M5	193.63	99	0.040 (0.031–0.048)	0.956	0.947	0.051	30,661.468	30,895.120	Second-order four-factor enhance and suppression strategies as higher-order factors
M6	193.07	99	0.040 (0.031–0.048)	0.956	0.947	0.051	30,660.447	30,894.099	Second-order four-factor positive ER ability and negative ER ability as higher-order factors
M7	234.71	100	0.047 (0.039–0.055)	0.951	0.941	0.051	30,660.572	30,889.816	One hierarchical second-order factor
M8	112.52	88	0.021 (0.005–0.032)	0.989	0.985	0.030	30,585.421	30,867.567	Bifactor CFA with one general factor and four specific ER ability

Note. SBχ^2^ = Satorra–Bentler scaled chi-square; *df* = degrees of freedom; CFI = Comparative Fit Index; TLI = Tucker–Levis Index; RMSEA = Root Mean Square Error of Approximation; 90% CI = 90% Confidence Interval for the RMSEA; SRMR = Standardized Root Mean Square Residual; AIC = Akaike Information Criterion; BIC = Bayesian Information Criterion.

**Table 3:** Standardized regression coefficients for enhancement and suppression abilities predicting mental health outcomes and relationship stress.

			DES			Anxiety				Stress			Relationship Stress			
	Step 1	Step 2	Step 3	Step 4	Step 1	Step 2	Step 3	Step 4	Step 1	Step 2	Step 3	Step 4	Step 1	Step 2	Step 3	Step 4
Gender	−0.15 **	−0.22 **	−0.22 **	−0.22 **	−0.08	−0.16 **	−0.16 **	−0.16 **	−0.06	−0.13 **	−0.14 **	−0.14 **	−0.08	−0.15 **	−0.15 **	−0.15 **
Age	0.07	0.04	0.04	0.04	0.10 *	0.07	0.07	0.07	0.10 *	0.06	0.05	0.05	0.08	0.03	0.03	0.03
ER-SEF		−0.31 **	−0.32 **	−0.32 **		−0.33 **	−0.34 **	−0.34 **		−0.36 **	−0.36 **	−0.36 **		−0.38 **	−0.36 **	−0.36 **
Enhancement			−0.06	−0.06			0.01	0.01			0.09	0.09			−0.26	−0.26
Suppression			0.08	0.07			0.03	0.02			−0.08	−0.08			0.21	0.21
EH*SUP				0.01				0.01				0.01				0.01
ΔR^2^	0.03 **	0.09 **	<0.001	<0.001	0.02 *	0.10 **	0.001	<0.001	0.02 *	0.12 **	<0.001	<0.001	0.01 *	0.13 **	0.01	<0.001
Total R^2^	0.03	0.12	0.13	0.13	0.02	0.13	0.13	0.13	0.02	0.14	0.14	0.14	0.01	0.15	0.15	0.15

Note. ER-SEF = emotion regulation self-efficacy; EH*SUP = interaction of enhancement and suppression. * *p* < .05, ** *p* < .01.

**Table 4:** Standardized regression coefficients for positive and negative abilities predicting mental health outcomes and relationship stress.

		Depression				Anxiety				Stress			Relationship Stress			
	Step 1	Step 2	Step 3	Step 4	Step 1	Step 2	Step 3	Step 4	Step 1	Step 2	Step 3	Step 4	Step 1	Step 2	Step 3	Step 4
Gender	−0.15 **	−0.22 **	−0.20 **	−0.20 **	−0.08	−0.16 **	−0.14 *	−0.14 *	−0.06	−0.13 **	−0.12 *	−0.12 *	−0.08	−0.15 **	−0.13 **	−0.13 **
Age	0.07	0.04	0.03	0.02	0.10 *	0.07	0.06	0.06	0.10 *	0.06	0.05	0.05	0.08	0.03	0.02	0.02
ER−SEF		−0.31 **	−0.29 **	−0.28 **		−0.33 **	−0.32 **	−0.32 **		−0.36 **	−0.34 **	−0.34 **		−0.38 **	−0.33 **	−0.33 **
PA			−0.54 **	−0.56 **			−0.43 *	−0.44 *			−0.46 *	−0.47 *			−0.66 **	−0.66 **
NA			0.56 **	0.57 **			0.46 *	0.47 *			0.46 **	0.48 **			0.61 *	0.62 *
PA*NA				−0.02				−0.02				−0.02				−0.01
ΔR^2^	0.03 **	0.09 **	0.02 *	0.001	0.02 *	0.10 **	0.02 *	<0.001	0.02 *	0.12 **	0.02 *	<0.001	0.01 *	0.13 **	0.03 **	<0.001
Total R^2^	0.03	0.12	0.15	0.15	0.02	0.13	0.14	0.14	0.02	0.14	0.15	0.15	0.01	0.15	0.18	0.18

Note. ER-SEF = emotion regulation self-efficacy; PA = positive expression regulation ability; NA = negative expression regulation ability; PA*NA = interaction of positive expression regulation ability and negative expression regulation ability. * *p* < .05, ** *p* < .01.

**Table 5:** Standardized regression coefficients for flexibility of enhancement and suppression abilities predicting mental health outcomes and relationship stress.

	Depression				Anxiety			Stress		Relationship Stress		
	Step 1	Step 2	Step 3	step1	Step 2	Step 3	Step 1	Step 2	Step 3	Step 1	Step 2	Step 3
Gender	−0.15 **	−0.22 **	−0.22 **	−0.08	−0.16 **	−0.16 **	−0.06	−0.13 **	−0.13 **	−0.07	−0.16 **	−0.16 **
Age	0.08	0.04	0.04	0.10 *	0.06	0.06	0.10 *	0.05	0.05	0.08	0.03	0.03
ER-SEF		−0.31 **	−0.31		−0.33 **	−0.33 **		−0.35 **	−0.35		−0.37 **	−0.36 **
Flexibility			0.01			0.01			−0.02			−0.04
ΔR^2^	0.03 **	0.09 **	<0.001	0.02 **	0.10 **	<0.001	0.02 *	0.12 **	<0.001	0.02 *	0.13 **	<0.003
Total R^2^	0.03	0.12	0.12	0.02	0.12	0.12	0.02	0.13	0.13	0.02	0.15	0.15

Note. ER-SEF = emotion regulation self-efficacy. * *p* < .05, ** *p* < .01.

**Table 6:** Standardized regression coefficients for flexibility of positive and negative expressive abilities predicting mental health outcomes and relationship stress.

	Depression				Anxiety			Stress		Relationship Stress		
	Step 1	Step 2	Step 3	Step 1	Step 2	Step 3	Step 1	Step 2	Step 3	Step 1	Step 2	Step 3
Gender	−0.15 **	−0.22 **	−0.21 **	−0.08 †	−0.16 **	−0.15 **	−0.06	−0.13 **	−0.13 **	−0.07	−0.16 **	−0.15 **
Age	0.08	0.04	0.03	0.10 *	0.06	0.06	0.10 *	0.05	0.05	0.08	0.03	0.03
ER_SEF		−0.31 **	−0.31 **		−0.33 **	−0.33 **		−0.35 **	−0.34 **		−0.37 **	−0.36 **
Flexibility of PE			−0.08 †			−0.07			−0.08 †			−0.11 *
Flexibility of NE			0.09 *			0.08 †			0.06			0.06
ΔR^2^	0.03 **	0.09 **	0.01 †	0.02 **	0.10 **	0.01	0.02 *	0.12 **	0.01	0.02 *	0.13 **	0.01 *
Total R^2^	0.03	0.12	0.13	0.02	0.12	0.13	0.02	0.13	0.14	0.02	0.15	0.16

Note. ER-SEF = emotion regulation self-efficacy. PE = positive emotion expression regulation; NA = negative emotion expression regulation. † *p* < .10, * *p* < .05, ** *p* < .01.

## Data Availability

Due to ethical restrictions from the project, the data were not made publicly available. Data and study materials can be accessed upon reasonable request by contacting the corresponding author.
